# Supplementary material for: Single-molecule long-read sequencing reveals a conserved intact long RNA profile in sperm
Source: Nat Commun. 2021 Mar 1;12:1361. doi: 10.1038/s41467-021-21524-6 (PMC7921563; doi:10.1038/s41467-021-21524-6)
Supplement: Supplementary file 1 — Supplementary Information [file 41467_2021_21524_MOESM1_ESM.pdf]

Supplementary Information

Single-molecule long-read sequencing reveals a conserved intact long RNA profile in sperm

Yu H. Sun, Anqi Wang, Chi Song, Goutham Shankar, Rajesh K. Srivastava, Kin Fai Au, Xin Zhiguo Li

Correspondence:
kinfai.au@osumc.edu (K.F.A.) & Xin_Li@URMC.rochester.edu (X.Z.L.)

This PDF file includes:

- Supplementary Figs. 1-5
- Supplementary Table 1
- Supplementary Table 2
- Supplementary Table 3

Supplementary Figures

a

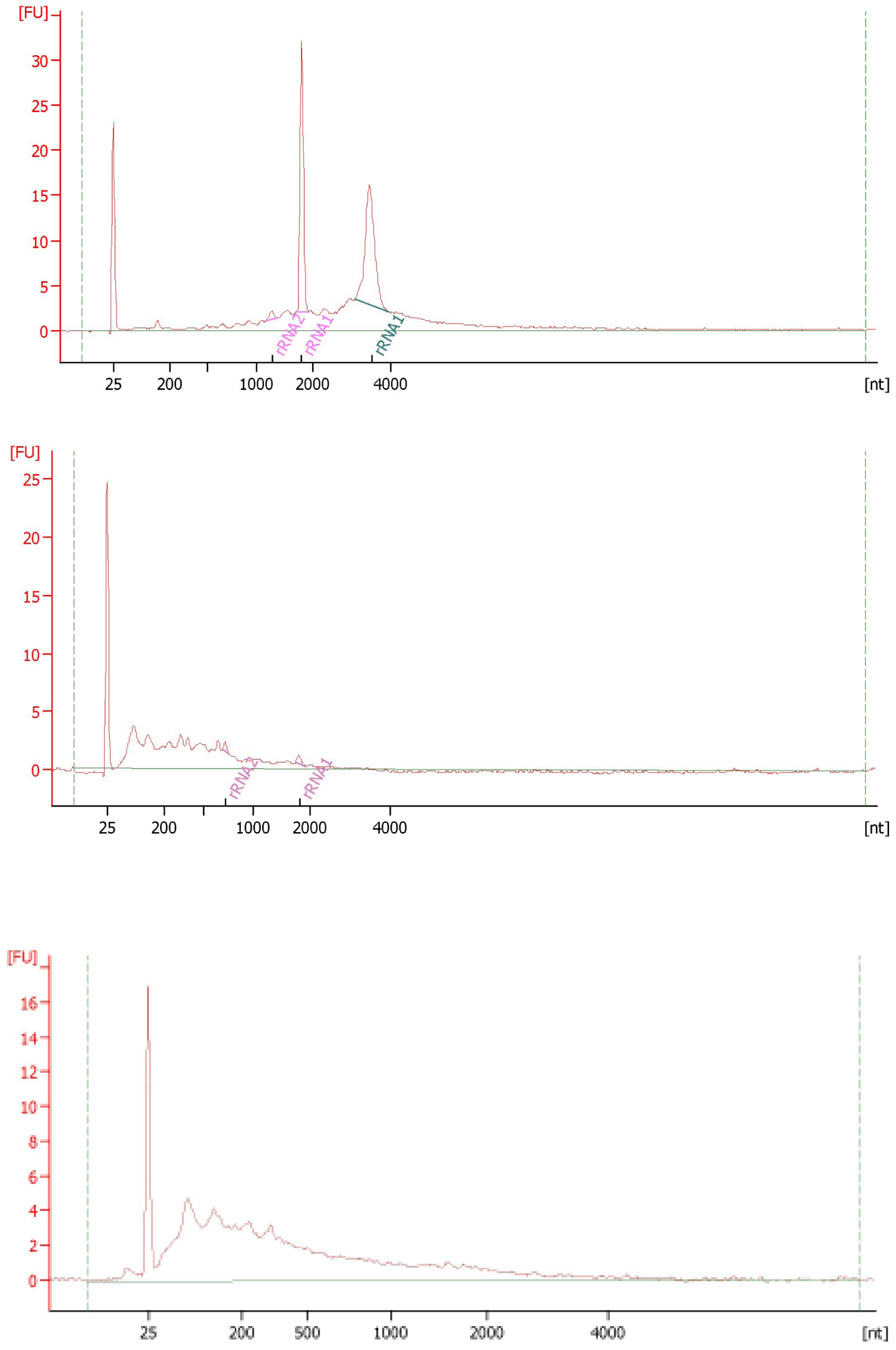

Mouse testis

Mouse sperm

Human sperm

b

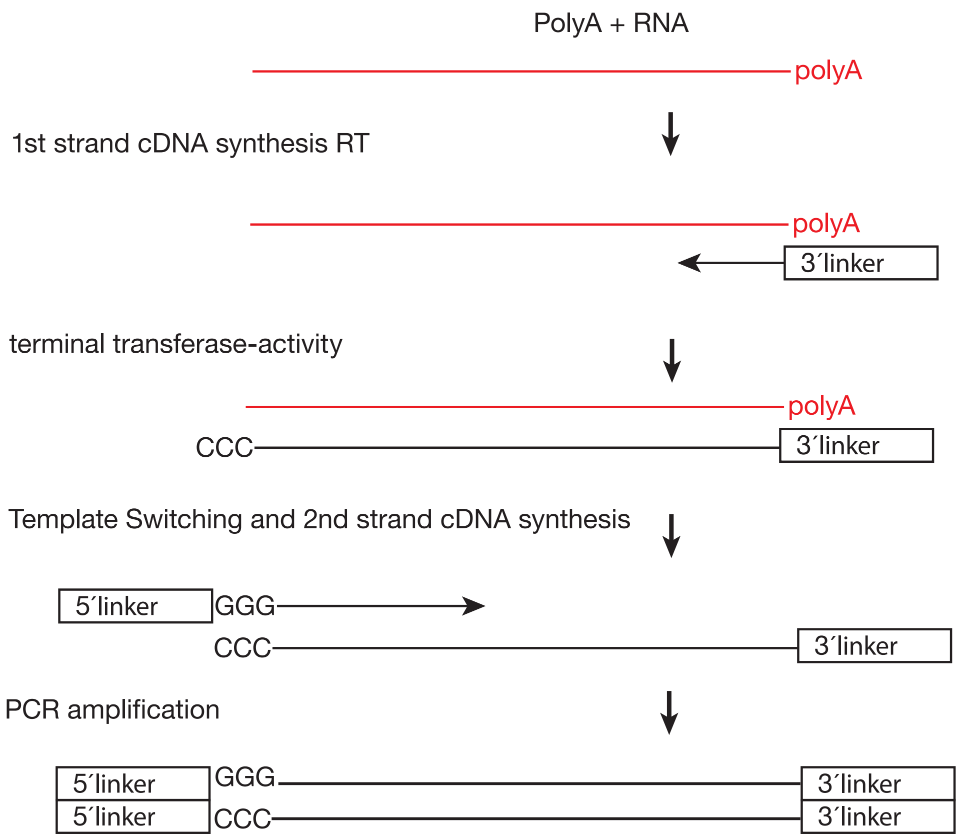
PolyA + RNA
polyA
1st strand cDNA synthesis RT
polyA
3´linker
terminal transferase-activity
polyA
CCC
3´linker
Template Switching and 2nd strand cDNA synthesis
5´linker
GGG
CCC
3´linker
PCR amplification
5´linker
GGG
3´linker
5´linker
CCC
3´linker

c

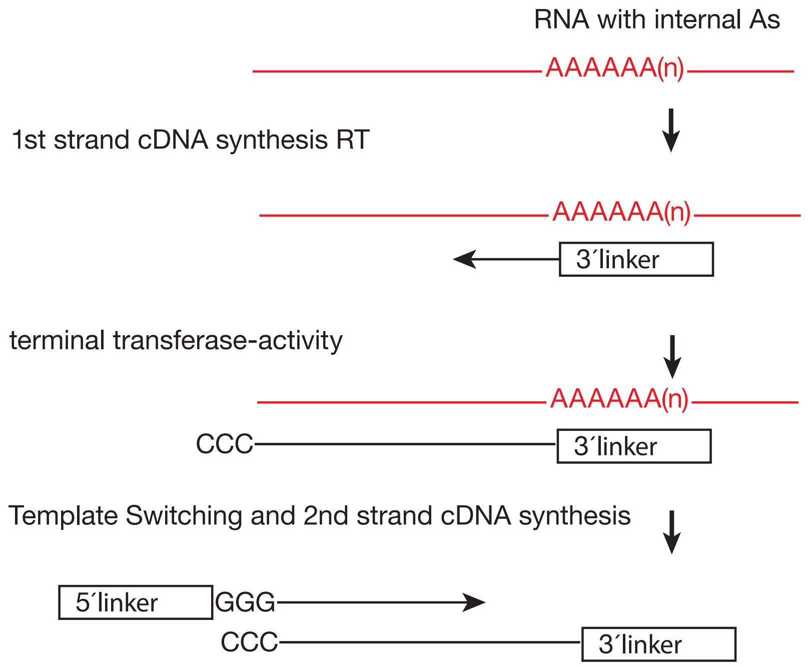
RNA with internal As
AAAAAA(n)
1st strand cDNA synthesis RT
AAAAAA(n)
3´linker
terminal transferase-activity
AAAAAA(n)
CCC
3´linker
Template Switching and 2nd strand cDNA synthesis
5´linker
GGG
CCC
3´linker

d

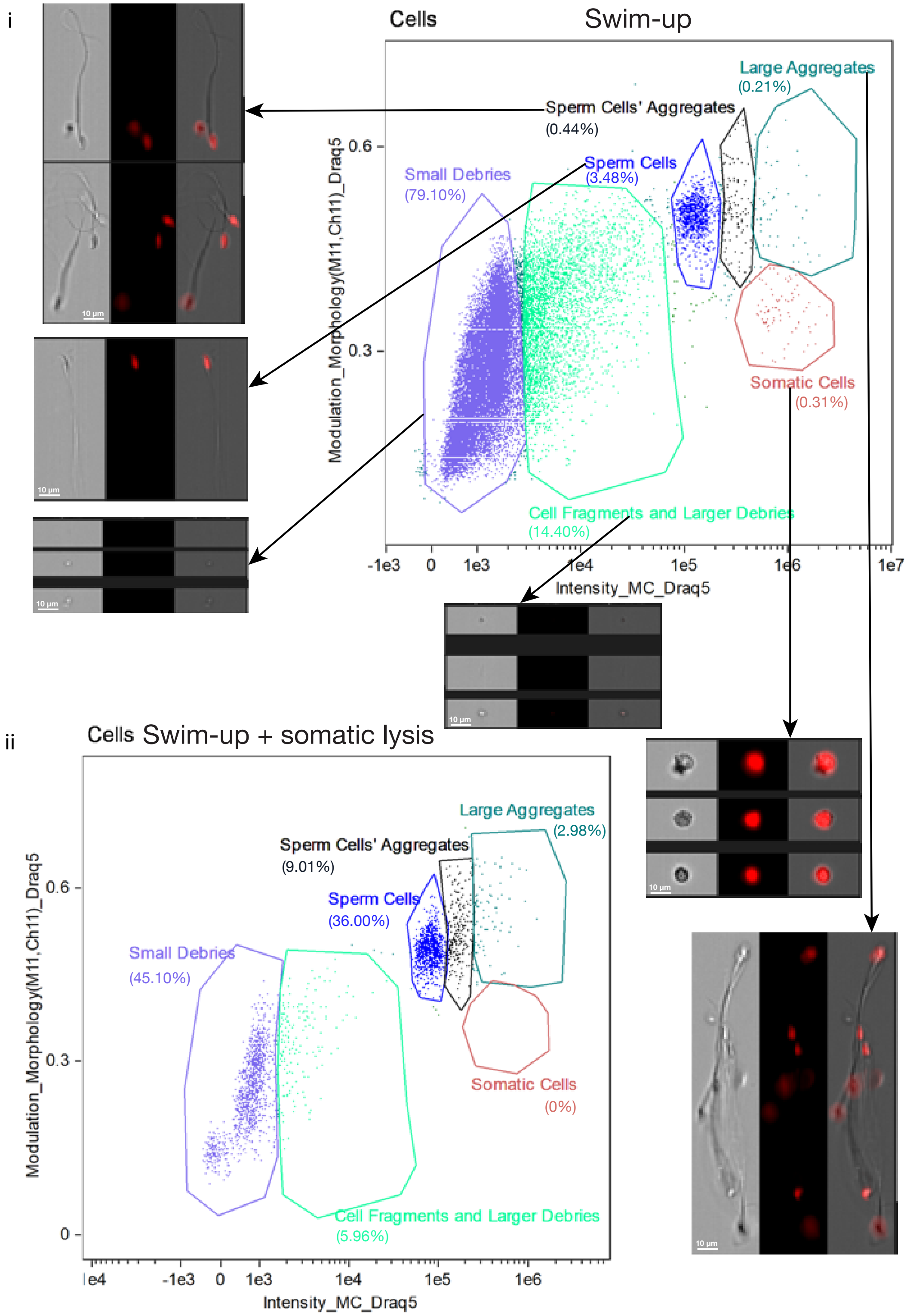
i
Cells
Swim-up
Large Aggregates
(0.21%)
Sperm Cells' Aggregates
(0.44%)
Sperm Cells
(3.48%)
Small Debries
(79.10%)
Somatic Cells
(0.31%)
Cell Fragments and Larger Debries
(14.40%)
Modulation_Morphology(M11,Ch11)_Draq5
Intensity_MC_Draq5
ii
Cells Swim-up + somatic lysis
Large Aggregates
(2.98%)
Sperm Cells' Aggregates
(9.01%)
Sperm Cells
(36.00%)
Small Debries
(45.10%)
Somatic Cells
(0%)
Cell Fragments and Larger Debries
(5.96%)
Modulation_Morphology(M11,Ch11)_Draq5
Intensity_MC_Draq5

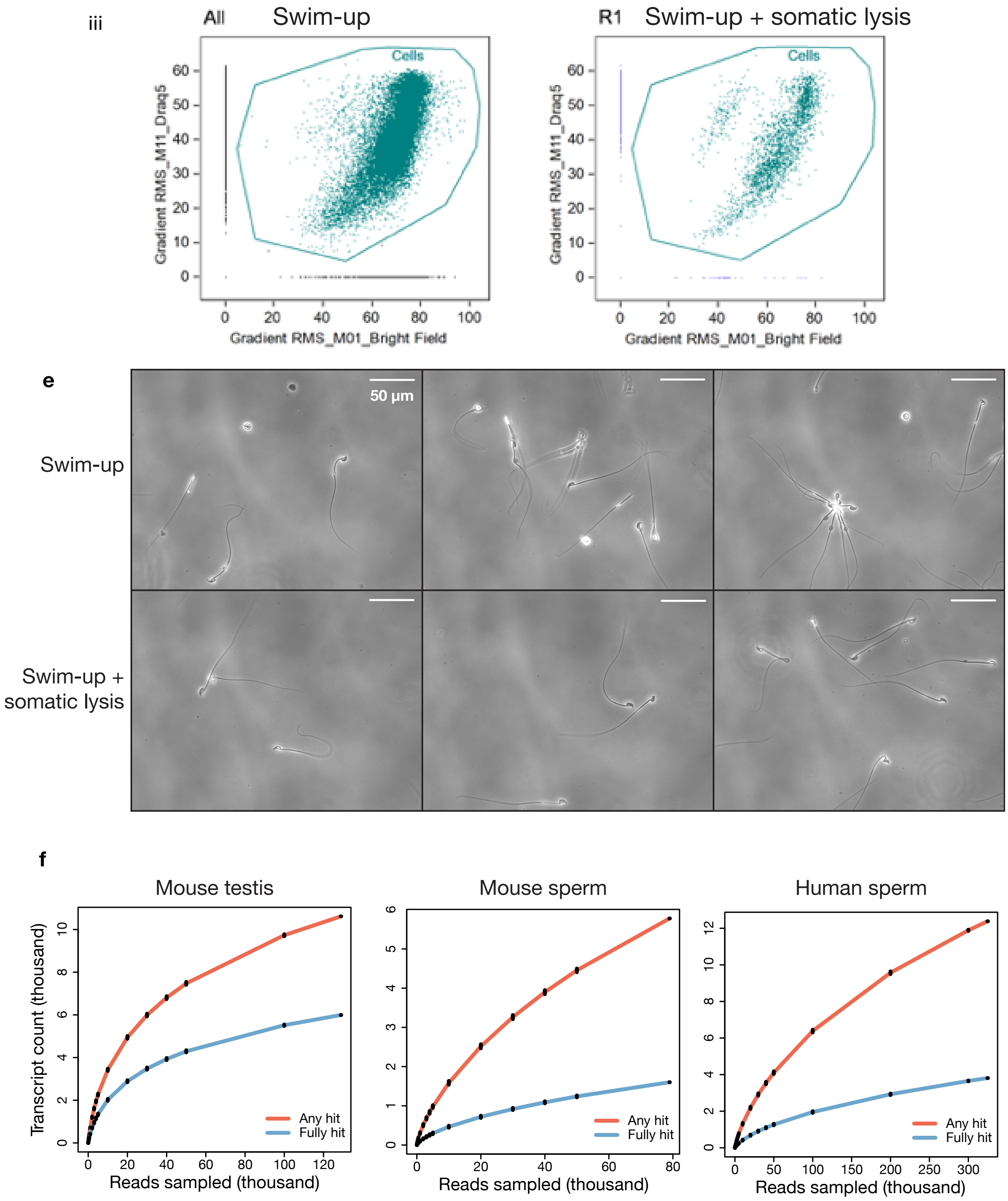
iii
All
Swim-up
Cells
Gradient RMS_M11_Draq5
Gradient RMS_M01_Bright Field
R1
Swim-up + somatic lysis
Cells
Gradient RMS_M11_Draq5
Gradient RMS_M01_Bright Field
e
50 µm
Swim-up
Swim-up + somatic lysis
f
Mouse testis
Mouse sperm
Human sperm
Transcript count (thousand)
Reads sampled (thousand)
Any hit
Fully hit

Supplementary Fig. 1. Defining intact transcripts in mouse sperm. a) Bioanalyzer profiles of mouse testis, mouse sperm, and human sperm. FU: fluorescence units. b) Iso-Seq library construction strategy. c) Potential artifacts that occur during Iso-Seq library construction due to internal priming. d) Flow cytometry analysis of mouse sperm released from cauda epididymis before (upper, i) and after the somatic lysis purification procedure (lower, ii). To analyze the data obtained from ImageStream, first most out of focus images were excluded by plotting events' Gradient RMS for bright field channel versus Gradient RMS for Draq5 channel (iii, The Gradient RMS feature measures the sharpness quality of an image by detecting large changes of pixel values in the image). Experiments were repeated at least three times independently with similar results. e) Microscopy analysis of mouse sperm released from cauda epididymis before (upper) and after the somatic lysis purification procedure (lower). Experiments were repeated at least three times independently with similar results. f) Transcript detection of long-read sequencing from mouse testis, mouse sperm, and human sperm. Rarefaction plot shows the number of transcripts detected (*y*-axis) as more reads were added to the analysis (*x*-axis). Different numbers of reads were randomly sampled from the successfully aligned reads, and the number of transcripts was calculated from the sampled reads. The black bar at each read count represents the range of detected transcripts from multiple sampling tests; the solid lines concatenate medians. The blue solid line represents the full-length matched transcripts only; red solid line represents transcripts detected with any match (full-length or partial). The fewer transcripts detected per increased number of reads sampled (i.e., the more the line flattens), the closer the analysis is to achieving complete detection (100% sensitivity). Source data of Supplementary Fig. 1e are provided as a Source Data file.

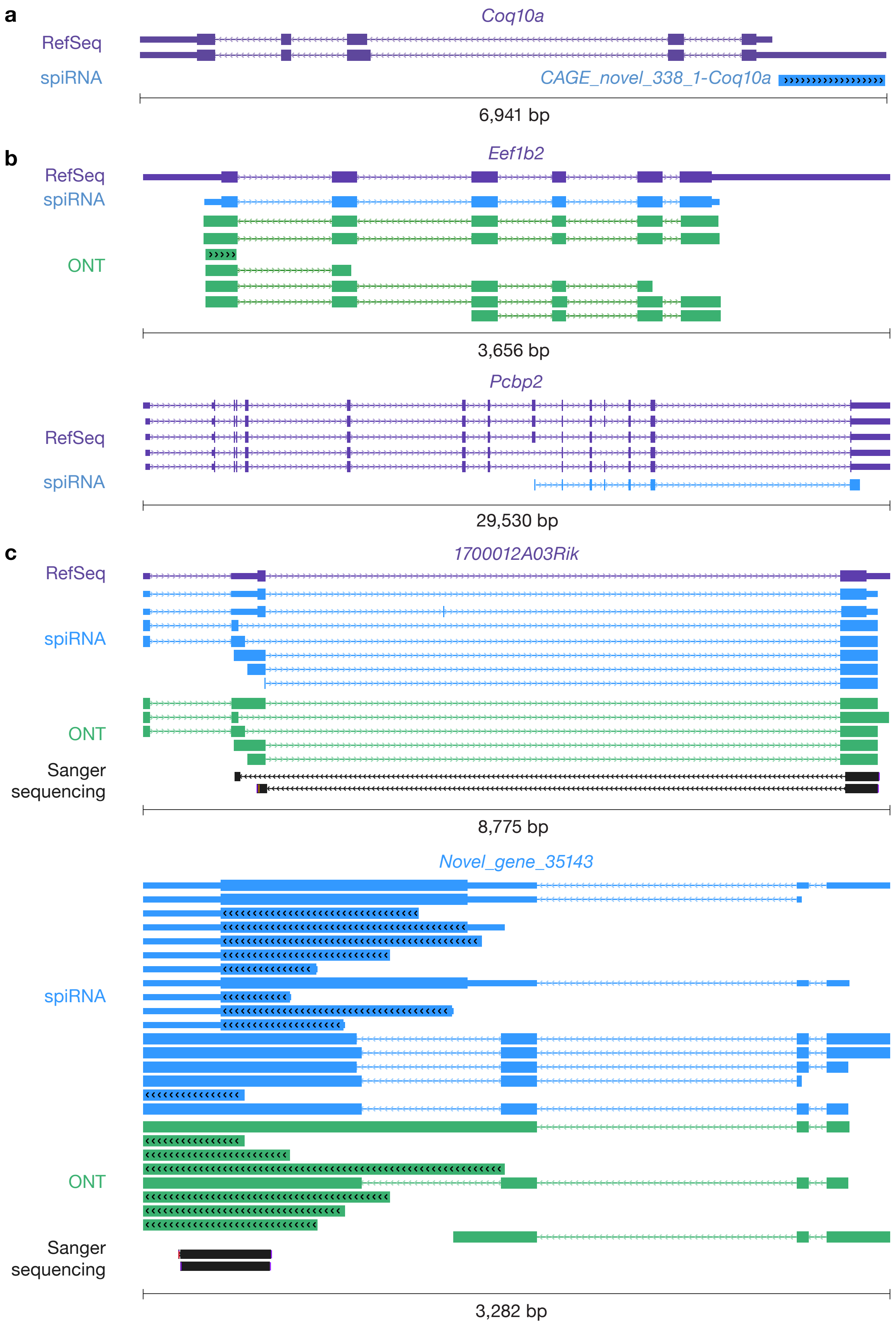
a
Coq10a
RefSeq
spiRNA
CAGE_novel_338_1-Coq10a
6,941 bp
b
Eef1b2
RefSeq
spiRNA
ONT
3,656 bp
Pcbp2
RefSeq
spiRNA
29,530 bp
c
1700012A03Rik
RefSeq
spiRNA
ONT
Sanger sequencing
8,775 bp
Novel_gene_35143
spiRNA
ONT
Sanger sequencing
3,282 bp

d

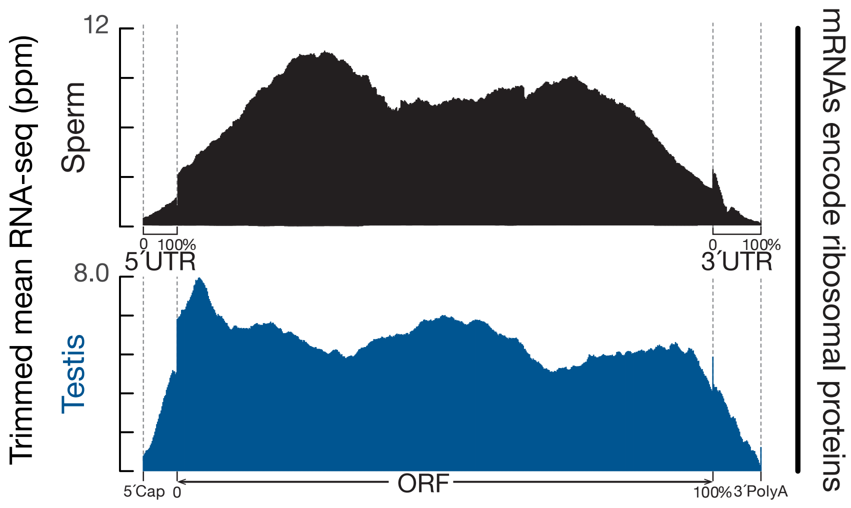

Supplementary Fig. 2. Intact transcripts found in mouse sperm. a) An example of a novel transcript that is antisense to a known gene locus. From top to bottom, RefSeq, and spiRNA. b) Two examples of novel transcript structures from known genes, *Eef1b2* and *Pcbp2*. From top to bottom, RefSeq and spiRNA. c) A novel isoform from *1700012A03Rik* Gene (upper) and a novel transcript from an intergenic region were confirmed by Sanger sequencing and ONT (Oxford Nanopore Technologies) sequencing. From top to bottom, RefSeq, spiRNAs, ONT sequencing reads, and the Sanger sequencing reads. d) Aggregated data for RNA-seq abundance on ribosomal protein-encoding mRNAs from sperm (*top*), and from testis (*bottom*) across 5´UTRs, ORFs, and 3´-UTRs. The x-axis represents the median length of these regions, and the y-axis represents the 10% trimmed mean of relative abundance. Ppm, parts per million.

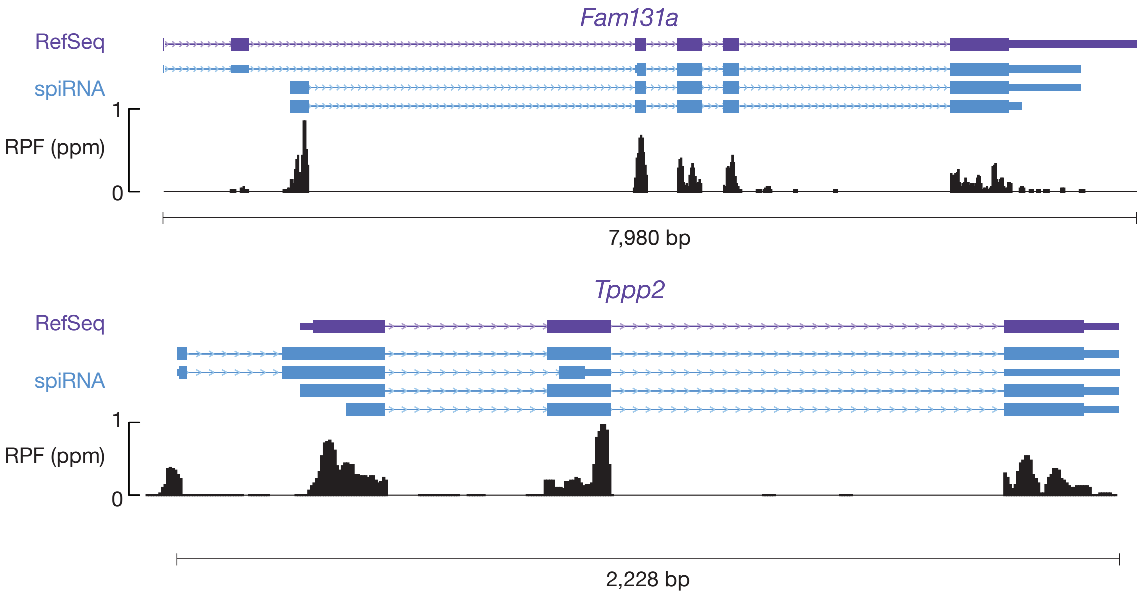

Supplementary Fig. 3. Novel spiRNAs are translated during spermatogenesis. Two examples of novel spiRNA transcript structures with altered coding regions in comparison to RefSeq annotation. From top to bottom, RefSeq, spiRNA, and RPF (ribosome protected fragment) reads.

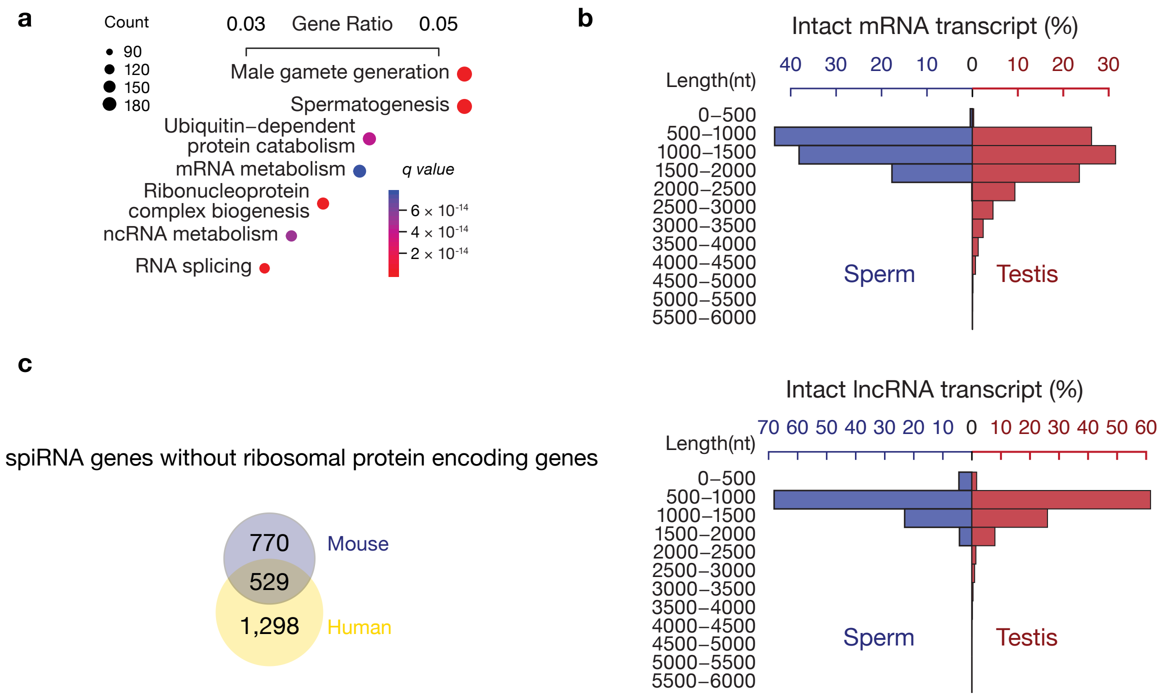

Supplementary Fig. 4. Conserved spiRNA profiles. a) GO-term enrichment analysis for biological pathway of testicular intact RNA genes. One-sided hypergeometric test was used to determine the *p* value. *q* value, adjusted *p* value using benjamini-hochberg correction. The plot displays the top 7 pathways by gene ratio (number of genes related to GO term / total number of genes associated with a GO term in mouse genome). b) Histogram showing transcript lengths of mRNAs (*top*) and lncRNAs (*bottom*). Blue, spiRNAs. Red, intact testicular long RNAs. c) Venn diagrams showing the overlapping gene loci of spiRNAs from mice and humans that exclude the mRNAs encoding ribosomal proteins. Source data of Supplementary Fig. 4b are provided as a Source Data file.

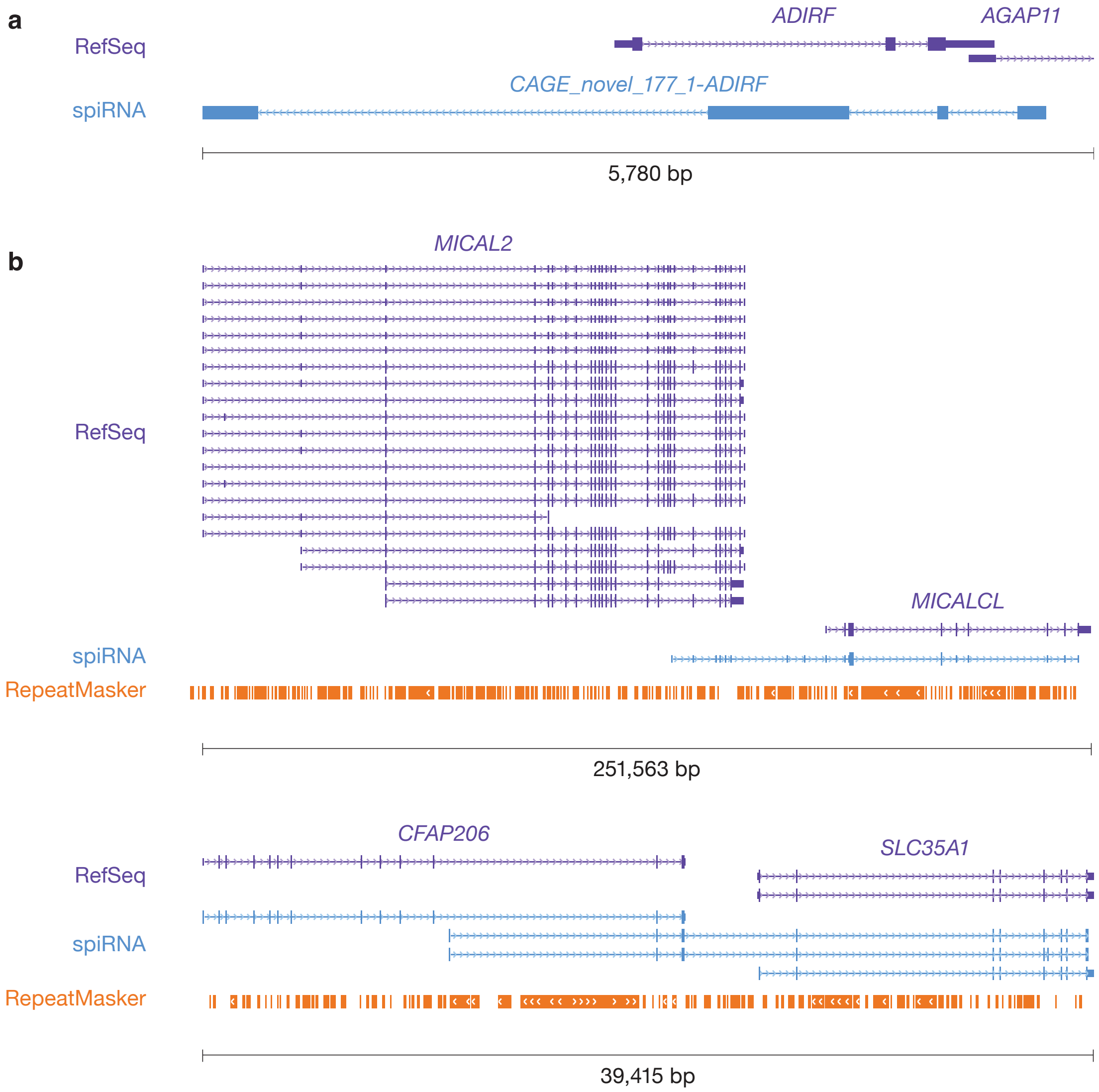

Supplementary Fig. 5. Novel intact transcripts in human sperm. a) An example of a novel transcript that is antisense to a known gene locus. From top to bottom, RefSeq and spiRNA. b) Two spiRNAs span neighboring annotated genes. From top to bottom, RefSeq, spiRNA and RepeatMasker.

Supplementary Table 1. Statistics of flow cytometry results

Gated cell types	Swim-up method		Swim-up + somatic lysis method	
	Count	% Gated	Count	% Gated
Sperm Cells	870	3.48	943	36.00
Small Debris	19,781	79.10	1,182	45.10
Cell Fragments and Larger Debris	3,598	14.40	156	5.96
Sperm Cells' Aggregates	111	0.44	236	9.01
Large Aggregates	52	0.21	78	2.98
Somatic Cells	78	0.31	0	0

Supplementary Table 2. Mouse sperm purity quantification

Template	qPCR quantification of *Myh11*
Epididymis cDNA	1.26×10^{-2}
1/10 Epididymis cDNA	5.79×10^{-3}
1/100 Epididymis cDNA	6.80×10^{-4}
1/1,000 Epididymis cDNA	3.85×10^{-5}
1/10,000 Epididymis cDNA	Not detected
Purified sperm cDNA	Not detected

Supplementary Table 3. PCR primers

F: Forward primer, R: Reverse primer

Detects	Species	Primer id	F/R	Sequences
Rps6 T7 Template	mouse	17.0105	F	CGTAATACGACTCACTATAGGGCTCGGCTGTGTCAAGATGAA
		17.0106	R	TAGAAGCTCTCAGTGAGGACAG
Rps6 qPCR	mouse	17.0092	F	AAGAAGATGATGTCCGCCAG
		17.0093	R	CAAGTCGCTGAATCTTGGGT
Rps8 T7 Template	mouse	20.0009	F	CGTAATACGACTCACTATAGGGCCTACCACAAGAAGCGAAAGTA
		20.0010	R	CCTTTCCGGGCTTTGATCTT
Rps8 qPCR	mouse	20.0019	F	CGAGTTCGAGGAGGCAATAAG
		20.0020	R	CGTTGTTGGATGCATTGTAGAC
Rpl11 T7 Template	mouse	20.0011	F	CGTAATACGACTCACTATAGGGCGGTGTTCTCCAAAGCTAGATAC
		20.0012	R	CTACCCAGCACCACATAGAAG
Rpl11 qPCR	mouse	20.0015	F	CTGAAGGTGCGGGAGTATG
		20.0016	R	CCAATGCTTGGGTCGTATTTG
Myh11 qPCR	mouse	15.0350	F	CTCTCCATCCGGTGTCCTC
		15.0351	R	TTCTCATCATCGCTGAGCTG
