# Supplementary material for: Single-molecule long-read sequencing reveals a conserved intact long RNA profile in sperm
Source: Nat Commun. 2021 Mar 1;12:1361. doi: 10.1038/s41467-021-21524-6 (PMC7921563; doi:10.1038/s41467-021-21524-6)
Supplement: Supplementary file 5 — Description of Additional Supplementary Files [file 41467_2021_21524_MOESM5_ESM.docx]

Description of Additional Supplementary files

Title: Supplementary Data 1.

Description: RNA quantification of bulk RNA sequencing from sperm and single-cell RNA-sequencing of epididymis in mice

Title: Supplementary Data 2.

Description: Sequencing Statistics (A) Mouse PacBio data statistics. (B) Mouse ONT data statistics. (C) Human PacBio data statistics. (D) Illumina RNA-seq data statistics (E) Mouse sperm intact RNAs (spiRNAs). (F) Mouse testis intact RNAs. (G) Human sperm intact RNAs (spiRNAs)
