# Supplementary material for: Single-molecule long-read sequencing reveals a conserved intact long RNA profile in sperm
Source: Nat Commun. 2021 Mar 1;12:1361. doi: 10.1038/s41467-021-21524-6 (PMC7921563; doi:10.1038/s41467-021-21524-6)
Supplement: Supplementary file 6 — Reporting Summary [file 41467_2021_21524_MOESM6_ESM.pdf]

Corresponding author(s): Xin Zhiguo Li

Last updated by author(s): Jan 11, 2021

Reporting Summary

Nature Research wishes to improve the reproducibility of the work that we publish. This form provides structure for consistency and transparency in reporting. For further information on Nature Research policies, see Authors & Referees and the Editorial Policy Checklist.

Statistics

For all statistical analyses, confirm that the following items are present in the figure legend, table legend, main text, or Methods section.

n/a	Confirmed	
☐	☒	The exact sample size (*n*) for each experimental group/condition, given as a discrete number and unit of measurement
☐	☒	A statement on whether measurements were taken from distinct samples or whether the same sample was measured repeatedly
☐	☒	The statistical test(s) used AND whether they are one- or two-sided<br>*Only common tests should be described solely by name; describe more complex techniques in the Methods section.*
☒	☐	A description of all covariates tested
☐	☒	A description of any assumptions or corrections, such as tests of normality and adjustment for multiple comparisons
☐	☒	A full description of the statistical parameters including central tendency (e.g. means) or other basic estimates (e.g. regression coefficient) AND variation (e.g. standard deviation) or associated estimates of uncertainty (e.g. confidence intervals)
☐	☒	For null hypothesis testing, the test statistic (e.g. *F*, *t*, *r*) with confidence intervals, effect sizes, degrees of freedom and *P* value noted<br>*Give P values as exact values whenever suitable.*
☒	☐	For Bayesian analysis, information on the choice of priors and Markov chain Monte Carlo settings
☒	☐	For hierarchical and complex designs, identification of the appropriate level for tests and full reporting of outcomes
☒	☐	Estimates of effect sizes (e.g. Cohen's *d*, Pearson's *r*), indicating how they were calculated

Our web collection on statistics for biologists contains articles on many of the points above.

Software and code

Policy information about availability of computer code

Data collection	Flow cytometry data was collected using ImageStream (Luminex Corporation, Austin, TX). Strand-specific single-end RNA sequencing (126 nt) was performed on Illumina HiSeq 2000 platform. Mouse sperm and testis PacBio data were sequenced using the PacBio RSII platform. We used PacBio Sequel platform to sequence human sperm data. Nanopore sequencing was performed on PromethION Beta.
Data analysis	Salmon (0.8.2), IDEAS (6.2), DART-PCR (1.0), SMRT Analysis pipeline (2.3.0), LoRDEC (0.5.3), GMAP (2014-12-24), HISAT2 (2.0.4), StringTie (1.3.1c), piPipes (1.4), Flexbar (2.2), TopHat (2.0.4), minimap2 (2.11-r797), R (3.5.0), GeneCycle package (1.1.4), clusterProfiler package (3.16.0). The codes for PacBio workflow are available at: https://github.com/LiLabZhaohua/PacBioWorkflow

For manuscripts utilizing custom algorithms or software that are central to the research but not yet described in published literature, software must be made available to editors/reviewers. We strongly encourage code deposition in a community repository (e.g. GitHub). See the Nature Research guidelines for submitting code & software for further information.

Data

Policy information about availability of data

All manuscripts must include a data availability statement. This statement should provide the following information, where applicable:

- Accession codes, unique identifiers, or web links for publicly available datasets
- A list of figures that have associated raw data
- A description of any restrictions on data availability

Next-generation sequencing data used in this study have been deposited at the NCBI Gene Expression Omnibus under the accession number: GSE137490 (https://www.ncbi.nlm.nih.gov/geo/query/acc.cgi?acc=GSE137490) and all relevant data are available from the authors. We also analyzed published datasets including GSE65786 (https://www.ncbi.nlm.nih.gov/geo/query/acc.cgi?acc=GSE65786), GSM1234252 (https://www.ncbi.nlm.nih.gov/geo/query/acc.cgi?acc=GSM1234252), GSM1088420 (https://www.ncbi.nlm.nih.gov/geo/query/acc.cgi?acc=GSM1088420), GSM1096581 (https://www.ncbi.nlm.nih.gov/geo/query/acc.cgi?acc=GSM1096581), GSM4030237 (https://www.ncbi.nlm.nih.gov/geo/query/acc.cgi?acc=GSM4030237), GSM4030238 (https://www.ncbi.nlm.nih.gov/geo/query/acc.cgi?acc=GSM4030238), GSM4030239 (https://www.ncbi.nlm.nih.gov/geo/query/acc.cgi?acc=GSM4030239), and GSM4030240 (https://www.ncbi.nlm.nih.gov/geo/query/acc.cgi?acc=GSM4030240), GSM1096580 (https://www.ncbi.nlm.nih.gov/geo/query/acc.cgi?acc=GSM1096580), GSM4030232 (https://

www.ncbi.nlm.nih.gov/geo/query/acc.cgi?acc=GSM4030232), GSM4030233 (https://www.ncbi.nlm.nih.gov/geo/query/acc.cgi?acc=GSM4030233), GSM4030234 (https://www.ncbi.nlm.nih.gov/geo/query/acc.cgi?acc=GSM4030234), GSM4030235 (https://www.ncbi.nlm.nih.gov/geo/query/acc.cgi?acc=GSM4030235), and GSM4030236 (https://www.ncbi.nlm.nih.gov/geo/query/acc.cgi?acc=GSM4030236). The source data underlying Figs 2e, 3b, 3e, 4b, 5d and Supplementary Figs 1f, 4b are provided as a Source Data file.

Field-specific reporting

Please select the one below that is the best fit for your research. If you are not sure, read the appropriate sections before making your selection.

☒ Life sciences ☐ Behavioural & social sciences ☐ Ecological, evolutionary & environmental sciences

For a reference copy of the document with all sections, see nature.com/documents/nr-reporting-summary-flat.pdf

Life sciences study design

All studies must disclose on these points even when the disclosure is negative.

Sample size	We use mice in congenic background to minimize the individual variation. Our prior experience demonstrates that three replicates provide superb statistical power in all relevant analyses.
Data exclusions	No data were excluded for analysis.
Replication	We include at least 3 biological replicates to compute our p value. All replicates were successful.
Randomization	Randomization procedures are not applicable to this study since we are not studying on population.
Blinding	Blinding is not relevant to our study as we don't have treatment or control groups.

Reporting for specific materials, systems and methods

We require information from authors about some types of materials, experimental systems and methods used in many studies. Here, indicate whether each material, system or method listed is relevant to your study. If you are not sure if a list item applies to your research, read the appropriate section before selecting a response.

Materials & experimental systems

n/a	Involved in the study	
☒	☐	Antibodies
☒	☐	Eukaryotic cell lines
☒	☐	Palaeontology
☐	☒	Animals and other organisms
☐	☒	Human research participants
☒	☐	Clinical data

Methods

n/a	Involved in the study	
☒	☐	ChIP-seq
☐	☒	Flow cytometry
☒	☐	MRI-based neuroimaging

Animals and other organisms

Policy information about studies involving animals; ARRIVE guidelines recommended for reporting animal research

Laboratory animals	We used C57BL/6J (Jackson Labs, Bar Harbor, ME, USA; stock number 664) mice. Sperm samples were acquired from 10-week to 6-month adult male mice. Mice were housed in a temperature (64-79°F) and humidity (30-70%) controlled room with 12 light/12 dark cycle.
Wild animals	The study did not involve wild animals.
Field-collected samples	The study did not involve sample collected from the wild.
Ethics oversight	Animals were maintained and used according to guidelines for animal care of the NIH and the University Committee on Animal Resources at the University of Rochester.

Note that full information on the approval of the study protocol must also be provided in the manuscript.

Human research participants

Policy information about studies involving human research participants

Population characteristics	Our criteria for taking the samples are rejecting for this study was the cutoff values of WHO Vth edition reference value, as

Population characteristics	defined for normozoospermic sample. If sample sperm count was more than 15 million/ml and motility was more than 40% it was accepted, otherwise rejected. All samples were from male patients who are less than 35 years old.
Recruitment	There was no recruitment for the patients for this purpose, since it was essentially an IRB exempt study.
Ethics oversight	University of Rochester Medical Center Review Board Protocol 00003599. We never obtain a consent for semen samples that are for diagnostic semen analysis. When IRB designates a sample as discarded it is exempt from the process of consenting. As mentioned in "Human Research Participant" section, we used only those samples that were normozoospermic as per WHO Vth Edition criterion. Before initiating the study, we got the IRB approval and it was determined that these samples are considered discarded samples. IRB approval number is already mentioned in the relevant section. Primary semen collection cup and any secondary tubes/containers were deidentified before we used the sample for this research purpose.

Note that full information on the approval of the study protocol must also be provided in the manuscript.

Flow Cytometry

Plots

Confirm that:

☒ The axis labels state the marker and fluorochrome used (e.g. CD4-FITC).

☒ The axis scales are clearly visible. Include numbers along axes only for bottom left plot of group (a 'group' is an analysis of identical markers).

☒ All plots are contour plots with outliers or pseudocolor plots.

☒ A numerical value for number of cells or percentage (with statistics) is provided.

Methodology

Sample preparation	We fixed the adult sperm cells from mice following the published protocol by adding 16 µl of 50 mM EDTA pH 8 per milliliter of cell suspension (48 µl for 3 ml) so as to obtain a final concentration of 0.8 mM EDTA, and then to fix germ cells slowly adding 3 volumes of ice-cold 100% ethanol using vortex at low speed.
Instrument	The data ware collected using ImageStream-X two camera system (Luminex Corporation, Austin, TX).
Software	The analysis was done using IDEAS 6.2 software (Luminex Corporation, Austin, TX).
Cell population abundance	Swim-up method: Sperm Cells (870), Small Debris (19,781), Cell Fragments and Larger Debris (3,598), Sperm Cells' Aggregates (111), Large Aggregates (52), Somatic Cells (78).<br>Swim-up + somatic lysis method: Sperm Cells (943), Small Debris (1,182), Cell Fragments and Larger Debris (156), Sperm Cells' Aggregates (236), Large Aggregates (78), Somatic Cells (0).
Gating strategy	First, out-of-focus objects were eliminated using Gradient RMS for both Draq5 and Bright Field images. Then, by plotting total Draq5 intensity, to differentiate 1n sperm cells and 2n somatic cells, versus Draq5 channel Modulation feature measurement (intensity range of an image, normalized between 0 and 1, using "Morphology" mask) we were able to identify six general population which contained: small debris, cell fragments and larger debris, sperm cells, sperm cells aggregates, somatic cells, and larger aggregates.

☒ Tick this box to confirm that a figure exemplifying the gating strategy is provided in the Supplementary Information.
